# Biomarkers in premature calves with and without respiratory distress syndrome

**DOI:** 10.1111/jvim.16217

**Published:** 2021-07-05

**Authors:** Merve Ider, Amir Naseri, Mahmut Ok, Kamil Uney, Alper Erturk, Murat K. Durgut, Tugba M. Parlak, Nimet Ismailoglu, Muhammed M. Kapar

**Affiliations:** ^1^ Faculty of Veterinary Medicine, Department of Internal Medicine Selcuk University Konya Turkey; ^2^ Faculty of Veterinary Medicine, Department of Pharmacology and Toxicology Selcuk University Konya Turkey; ^3^ Faculty of Veterinary Medicine, Department of Internal Medicine Mustafa Kemal University Hatay Turkey

**Keywords:** endothelial biomarkers, premature calf, pulmonary epithelium, pulmonary hypertension, RDS

## Abstract

**Background:**

Approaches to the evaluation of pulmonary arterial hypertension (PAH) in premature calves by using lung‐specific epithelial and endothelial biomarkers are needed.

**Objective:**

To investigate the evaluation of PAH in premature calves with and without respiratory distress syndrome (RDS) by using lung‐specific epithelial and endothelial biomarkers and determine the prognostic value of these markers in premature calves.

**Animals:**

Fifty premature calves with RDS, 20 non‐RDS premature calves, and 10 healthy term calves.

**Methods:**

Hypoxia, hypercapnia, and tachypnea were considered criteria for RDS. Arterial blood gases (PaO_2_, PaCO_2_, oxygen saturation [SO_2_], base excess [BE], and serum lactate concentration) were measured to assess hypoxia. Serum concentrations of lung‐specific growth differentiation factor‐15 (GDF‐15), asymmetric dimethylarginine (ADMA), endothelin‐1 (ET‐1), vascular endothelial growth factor (VEGF), and surfactant protein D (SP‐D) were measured to assess PAH.

**Results:**

Arterial blood pH, PaO_2_, SO_2_, and BE of premature calves with RDS were significantly lower and PaCO_2_ and lactate concentrations higher compared to non‐RDS premature and healthy calves. The ADMA and SP‐D concentrations of premature calves with RDS were lower and serum ET‐1 concentrations higher than those of non‐RDS premature and healthy calves. No statistical differences for GDF‐15 and VEGF were found among groups.

**Conclusions and Clinical Importance:**

Significant increases in serum ET‐1 concentrations and decreases in ADMA and SP‐D concentrations highlight the utility of these markers in the diagnosis of PAH in premature calves with RDS. Also, we found that ET‐1 was a reliable diagnostic and prognostic biomarker for PAH and predicting mortality in premature calves.

**Abbreviations**
ADMA: asymmetric dimethylarginine
ARDS: acute respiratory distress syndrome
BE: base excess
ET‐1: endothelin‐1
FiO_2_
: fraction of inspired oxygen
GDF‐15: lung‐specific growth differentiation factor‐15
iNOS: nitric oxide synthase
NO: nitric oxide
non‐RDS: without respiratory distress syndrome
PaCO_2_
: partial pressure of carbon dioxide
PAH: pulmonary arterial hypertension
PaO_2_, PHT: pulmonary hypertension; partial pressure of oxygen
RDS: respiratory distress syndrome
ROC: receiver operating characteristic curve
SO_2_
: oxygen saturation
SP‐D: surfactant protein‐D
VEGF: vascular endothelial growth factor

## 1 INTRODUCTION

In cows and heifers, average perinatal mortality on dairy farms ranges from 2% to 20%, and in developed countries between 5% and 8%. Premature birth remains an important and common cause of calf mortality in the world.^1^, ^2^, ^3^, ^4^ The most important problems in premature infants is respiratory distress syndrome (RDS) that leads to increased respiratory effort and inadequate oxygen exchange.^5^, ^6^, ^7^, ^8^ The RDS caused by surfactant insufficiency is associated with pulmonary hypertension (PHT) in lambs.^9^ In premature newborns, the lung lobes cannot inflate effectively because of surfactant deficiency, and hypoxia develops because gas exchange does not occur adequately. As a result, alveolar and interstitial edema develop related to interstitial inflammation and endothelial and epithelial damage. Developing hypoxia and alveolar and interstitial edema cause narrowing of the pulmonary arteries and therefore development of pulmonary arterial hypertension (PAH).^10^, ^11^, ^12^ Studies in premature infants have shown that the development of PAH substantially increases mortality.^13^, ^14^ Recently, in human medicine, a noninvasive method for determining PAH employs biomarkers specific to pulmonary epithelial and endothelial damage.^13^, ^15^, ^16^, ^17^, ^18^, ^19^

Growth differential factor‐15 (GDF‐15), asymmetric dimethylarginine (ADMA), endothelial‐1 (ET‐1), vascular endothelial growth factor (VEGF), and surfactant protein‐D (SP‐D) concentrations were found to change significantly in infants with PHT.^15^, ^18^, ^20^, ^21^, ^22^, ^23^ The concentration of GDF‐15 is used as a biomarker to evaluate the prognosis of PHT.^21^, ^23^ These biomarkers are used to determine the proliferation and apoptosis of endothelial cells in PHT cases.^23^ Asymmetric dimethylarginine is a naturally occurring amino acid that prevents production of nitric oxide (NO), an end product of oxidative stress. Increased concentrations of ADMA attenuate NO concentrations leading to an increase in vascular tone. Therefore, it has been suggested that ADMA can be considered a useful biomarker in PHT cases.^18^

Endothelial‐1 is a peptide abundantly present in the human lung and plays an important role in the development of PHT because of the presence of endothelin receptors on vascular smooth muscle cells.^13^ Earlier studies indicated that the concentration of ET‐1 in plasma and lung tissue is significantly increased in patients with PHT.^15^, ^22^ Vascular endothelial growth factor and its receptor concentrations were found to be increased with damage to pulmonary vessels in patients with PHT.^15^ Furthermore, VEGF concentrations also increased significantly during hypoxic conditions.^24^ Surfactant protein‐D is secreted by type II pneumocytes and plays an important role in ensuring and maintaining the surface integrity of alveoli. In patients with acute RDS (ARDS), SP‐D concentrations decreased with destruction of type II pneumocytes in the lungs depending on the severity of damage to the lung.^25^ The changes in the SP‐D concentration in ARDS patients could provide valuable information about the prognosis of the disease.^16^, ^26^

Therefore, we aimed to determine: (a) whether PAH had developed in premature calves with and without RDS by evaluating lung epithelial and endothelial damage biomarkers and (b) the importance of these biomarkers in predicting mortality in affected calves.

## 2 MATERIALS AND METHODS

### 2.1 Animals

The experimental groups consisted of 50 premature calves with RDS (28 Holstein, 19 Simmental, 2 Brown‐Swiss) and 20 premature calves without RDS (12 Holstein, 7 Simmental, 1 Brown‐Swiss) admitted to the Selcuk University Large Animal Hospital of the Faculty of Veterinary Medicine were enrolled in the study.

Ten healthy normal‐term calves (6 Holstein, 2 Simmental, 2 Brown‐Swiss) from the Faculty Farm also were examined. The gestational age range for premature calves was 230 to 255 days and >280 days for healthy calves.

### 2.2 Clinical evaluation

Upon admission of premature calves to the clinic, history was taken, and live weight, age, and breed were recorded. Routine clinical examinations of all calves were performed. According to clinical observations and blood gas analysis, premature calves that met the criteria for RDS were enrolled in the trial group, premature calves without RDS were enrolled in the positive control group, and healthy normal‐term calves were enrolled in the negative control group. The criteria for prematurity were decreased gestation period (<255 days), low body weight (BW), a short silky hair coat, incomplete eruption of incisors, soft hooves, and weak or no suckling reflex.^7^, ^8^, ^27^

### 2.3 Criteria for definition of RDS

Hypoxia (PaO_2_ < 60 mm Hg), hypercapnia (PaCO_2_ > 45 mm Hg), tachypnea (breaths per minute >45/min), and abdominal respiration with wheezing were the criteria for distinguishing between calves with or without RDS.^7^, ^8^, ^27^, ^28^ A PaO_2_ <60 mm Hg and at least 2 other criteria described above were required for a case to be diagnosed as RDS.

### 2.4 Collection of blood samples

Blood samples were collected from the calves for arterial blood gas analysis and lung‐specific biomarker measurements at the time of admission. For uniformity among the groups, all blood samples were taken within the first 12 hours after birth. Blood samples for serum were taken from jugular vein and for blood gas measurement from auricular arteries. Nonanticoagulant tubes were used for serum and sodium heparin‐containing plastic syringes were used for blood gas measurement. Blood samples taken for biochemical analyses were kept at room temperature for 15 minutes, then centrifuged at 2000*g* for 10 minutes. Sera were removed and stored at −80°C. Blood gas measurements were performed within 5 to 10 minutes of collection.

### 2.5 Blood gas analysis

Heparinized arterial blood pH, PaCO_2_, PaO_2_, oxygen saturation (SO_2_), base excess (BE), and lactate concentration were measured using a GEM Premier Plus 3000 analyzer (74351, Blood Gas/ Electrolyte Analyzer, Model 5700; Instrumentation Laboratories, Massachusetts).

### 2.6 Lung‐specific endothelial and epithelial biomarker analyses

Serum GDF‐15, ADMA, ET‐1, VEGF, and SP‐D concentration of all calves were measured using commercial bovine‐specific ELISA test kits according to the manufacturer's instructions (Bioassay Technology Laboratory, Shanghai, China): bovine growth differentiation factor ELISA kit (Bioassay Technology Laboratory, LOT:202006012), bovine asymmetrical dimethylarginine ELISA kit (Bioassay Technology Laboratory, LOT:202006012), bovine endothelin‐1 ELISA kit (Bioassay Technology Laboratory, LOT:202006012), bovine vascular endothelial cell growth factor ELISA kit (Bioassay Technology Laboratory, LOT:202006012), and bovine SP‐D ELISA kit (Bioassay Technology Laboratory, LOT:202006012). Intra‐assay coefficients of variation, inter‐assay coefficients of variation, and detectable ranges were ≤8%, ≤10%, and 7‐1500 ng/L for GDF‐15; ≤8%, ≤10%, and 0.05‐10 nmol/mL for ADMA; ≤8%, ≤10%, and 2‐600 ng/L for ET‐1; ≤8%, ≤10%, and 15‐3000 ng/L for VEGF; and ≤8%, ≤10%, and 1‐400 ng/L for SP‐D, respectively.

### 2.7 Treatment protocol

In premature calves without RDS, only the standard treatment protocol was applied whereas premature calves with RDS were treated using a standard treatment protocol along with oxygen application and nebulizer treatment.

#### 2.7.1 Standard treatment

Standard treatment consisted of 5 mL calcium (Calcio PH, Fatro, Istanbul, Turkey), 3 mL phosphorus (Metafos, Teknovet, Istanbul, Turkey) and 3 mL vitamin C (Vita‐C Vetoquinol, Novakim, Kocaeli, Turkey) q12h for 3 days, 10 mg/kg BW erythromycin (Erivet, Biomed, Istanbul, Turkey) q24h for 3 days, vitamins A, D, and E (Adesol AD3E, Topkim, Istanbul, Turkey), and selenium and vitamin E (Selephos, Topkim, Istanbul, Turkey) 1 mL once IM. A 1.3% solution of NaHCO_3_ (Carbotek, Teknovet, Istanbul, Turkey; 250‐500 mL) was administered to calves with base deficits, and 5% dextrose (5% Dextrose Mediflex, Istanbul, Turkey) solution (100‐350 mL) was administered to calves with hypoglycemia IV. In addition, calves received septiserum once SC (Septicol, Adıyaman, Turkey).

#### 2.7.2 Oxygen application

Oxygen was administered to premature calves with RDS. Oxygen was passed through water to humidify it and given to the calves using a suitable oxygen mask. Oxygen initially was administered intranasally 15 minutes at a flow rate of 5 to 6 L/min for 3 hours with a 10‐minute break after each 15 minutes. After 3 hours, the oxygen flow rate was decreased to 3 to 4 L/min. Oxygen treatment was continued until SO_2_ reached 80%.^7^, ^8^

#### 2.7.3 Nebulizer application

An ultrasonic nebulization device (NebuTech Brand SoHuMa II model, Elsenfeld, Germany) and combinations of formoterol, furosemide, and fluticasone were administered to calves with RDS at doses mentioned below. After each use and before use in a new patient, the device was cleaned, and sterilization procedures were performed in accordance with the procedure.

#### 2.7.4 Doses of inhalation drugs

The nebulization form of fluticasone (fluticasone propionate, Flixotide, GlaxoSmithKline, Istanbul, Turkey) was diluted with 2.5 mL saline solution and administered at a dosage of 15 μg/kg BW over 5 minutes by nebulization for 3 days with 12 hours between treatments.^7^, ^8^ The nebulization form of formoterol (Foradil, Novartis, Ankara, Turkey) was diluted with 2.5 mL saline solution and administered at a dosage of 12 μg/kg BW over 5 minutes by nebulization for 3 days with 12 hours between treatments.^8^ The parenteral form of furosemide (Desal, Munir Sahin İlac, Istanbul, Turkey) was diluted with 2.5 mL saline solution and administered at a dosage of 1 mg/kg BW over 5 minutes by nebulization for 3 days with 12 hours between treatments.^7^, ^8^

### 2.8 Statistical analysis

The statistics program SPSS 25 (IBM Corp. 2017) was used to evaluate the data. The Kolmogorov‐Smirnov test was used to determine the normality of variables and the homogeneity of variances. Parametric data were evaluated using one‐way analysis of variance (ANOVA) and the post hoc Tukey test using mean ± SD and nonparametric data were evaluated using the Mann‐Whitney *U* test as median (minimum/maximum). For detection of correlation between variables, Spearman correlation test and linear regression analysis were used. The prognostic value of ET‐1 was evaluated using receiver operating characteristic (ROC) curve analysis to determine the prognostic cutoffs for the best differentiation between survivors and nonsurvivors. *P* < .05 and *P* < .01 were considered significant.

## 3 RESULTS

### 3.1 Clinical findings

The mean weights of the calves were 22.32 ± 4.90 kg in the RDS group, 23.35 ± 3.70 kg in the non‐RDS calf group, and 44.60 ± 2.83 kg in the control group. Thirty‐five (70%) of the 50 calves in the RDS group, and 19 (95%) of the 20 calves in the non‐RDS group survived, whereas 15 calves in the RDS group and 1 calf in the non‐RDS group died within 48 hours.

Clinical findings such as short gestational period, low BW, teeth not fully separated from the gums, short and soft hair, soft hooves, weakness in muscles and tendons, and weak or no suckling reflex were observed in the premature calves. Additionally, apnea or tachypnea, abdominal or wheezing respirations, cyanotic or pale mucous membranes, prolonged capillary refill time, and hypothermia were present in premature calves with RDS.

Some calves in the RDS group also had abdominal distention after PO feeding and could not eliminate meconium without enema assistance. Treated premature calves had increased interest in the environment, attempted to maintain sternal position with support, and experienced decreased wheezing respirations within 24 hours. After 48 hours of treatment, increased suckling reflexes, efforts to stand up with support, and costo‐abdominal respiration were observed. After 72 hours, premature calves had good suckling reflex and were able to stand up and walk.

### 3.2 Blood gas analysis

Arterial blood gas parameters of premature and healthy calves are presented in Table 1. In the RDS group, pH, PaO_2_, SO_2_, and BE were significantly lower whereas PaCO_2_ and lactate concentrations were higher than in the non‐RDS and control groups. Compared to the control group, PaO_2_ was significantly lower (*P* < .05) in the non‐RDS group (Table 1).

**TABLE 1:** Arterial blood gas parameters of premature and healthy calves

Parameters	RDS group (n:50)	Non‐RDS group (n: 20)	Control group (n: 10)
pH	7.14 ± 0.16^B^	7.40 ± 0.66^A^	7.40 ± 0.43^A^
PaCO_2_ (mm Hg)	56.76 ± 11.19^A^	39.02 ± 4.48^B^	40.90 ± 4.33^B^
PaO_2_ (mm Hg)	28.76 ± 8.10^C^	52.11 ± 11.57^B^	63.80 ± 11.07^A^
SO_2_%	52.88 ± 23.59^B^	86.31 ± 9.48^A^	90.90 ± 4.33^A^
Lac (mmol/L)	7.30^A^ (3‐23)	2.95^BC^ (0.9‐9.20)	3.60^C^ (1.90‐6.9
BE (mmol/L) median/(min/max)	−6.50^A^ (−29.00 to 11.20)	−0.82^B^ (−9.50 to 5.70)	1.15^B^ (−3.70 to 6.30)

*Note*: Different letters in the same line are statistically significant (*P* < .05).

Abbreviations: BE, base deficit; Lac, lactate; PaCO_2_, arterial partial pressure of carbon dioxide; PaO_2_, arterial partial pressure of oxygen; SO_2_, oxygen saturation.

### 3.3 Biomarker analysis

Serum biomarker concentrations in the premature and healthy calves are presented in Table 2. Serum ADMA and SP‐D concentrations of the RDS group were found to be significantly lower than those of the control group (*P* < .05). Serum ET‐1 concentration of the RDS group were significantly higher than those of the non‐RDS and control groups (*P* < .05). No statistical differences (*P* > .05) were found among all groups for GDF‐15 and VEGF concentrations (Table 2). Correlations between arterial blood gas parameters and ADMA, ET‐1, and SP‐D concentrations in the premature and healthy calves are presented in Table 3. Positive correlations between blood pH and PaO_2_, SO_2_, and BE were found whereas a negative correlation was found among PaCO_2_, lactate, and ET‐1 concentrations (*P* < .01). A positive correlation between blood PaCO_2_ and lactate concentrations (*P* < .01) and ET‐1 (*P* < .05) and negative correlations among PaO_2_, SO_2_, and BE (*P* < .01) were identified. Positive correlations between blood PaO_2_ and SO_2_ (*P* < .01), BE (*P* < .05), and SP‐D (*P* < .05), and negative correlation between lactate and ET‐1 concentrations (*P* < .01) were found. A negative correlation between lactate concentration and BE and a positive correlation with ET‐1 concentration (*P* < .01) were found. A negative correlation was identified between blood BE and ET‐1 concentrations (*P* < .01).

**TABLE 2:** Biomarker concentrations measured in premature and healthy calves

Variables	RDS group (n: 50)	Non‐RDS group (n: 20)	Control group (n: 100)
ADMA (nmol/mL)	0.48 ± 0.09^B^	0.52 ± 0.06^AB^	0.56 ± 0.09^A^
ET‐1 (ng/L) median/(min/max)	18.25^A^ (0.08‐49.40)	7.59^BC^ (0.08‐25.88)	5.56^C^ (3.71‐12.12)
GDF‐15 (ng/L)	62.50 ± 12.94	68.14 ± 11.72	64.73 ± 9.36
SP‐D (ng/mL)	42.52 ± 11.40^B^	49.35 ± 6.87^AB^	51.28 ± 10.58^A^
VEGF (ng/L)	109.22 ± 20.02	116.83 ± 13.05	115.87 ± 19.26

*Note*: Different letters in the same line are statistically significant (*P* < .05).

Abbreviations: ADMA, asymmetric dimethylarginine; ET‐1, endothelin‐1; GDF‐15, growth differentiation faktor‐15; SP‐D, surfactant protein; VEGF, vascular endothelial growth factor.

**TABLE 3:** Correlation results between arterial blood gas parameters and ADMA, ET‐1, and SP‐D concentrations in premature and healthy calves (Pearson correlation analysis)

Parameters	pH	PaCO_2_	PaO_2_	SO_2_	Lac	BE	ADMA	ET‐1	SP‐D
pH	1	−0.76**	0.51**	0.63**	−0.80**	0.85**	0.09	−0.38**	0.16
PaCO_2_		1	−0.60**	−0.66**	0.52**	−0.40**	−0.05	0.26*	−0.08
PaO_2_			1	0.79**	−0.33**	0.27*	0.21	−0.31**	0.25*
SO_2_				1	−0.46**	0.39**	0.09	−0.44**	0.18
Lac					1	−0.82**	−0.09	0.38**	−0.17
BE						1	0.07	−0.37**	0.14
ADMA							1	−0.42**	0.82**
ET‐1								1	−0.44**
SP‐D									1

Abbreviations: ADMA, asymmetric dimethylarginine; BE, base deficit; ET‐1, endothelin‐1; Lac, lactate; PaCO_2_, arterial partial pressure of carbon dioxide; PaO_2_, arterial partial pressure of oxygen; SO_2_, oxygen saturation; SP‐D, surfactant protein.

* *P* < .05.

** *P* < .01.

**FIGURE 1:**
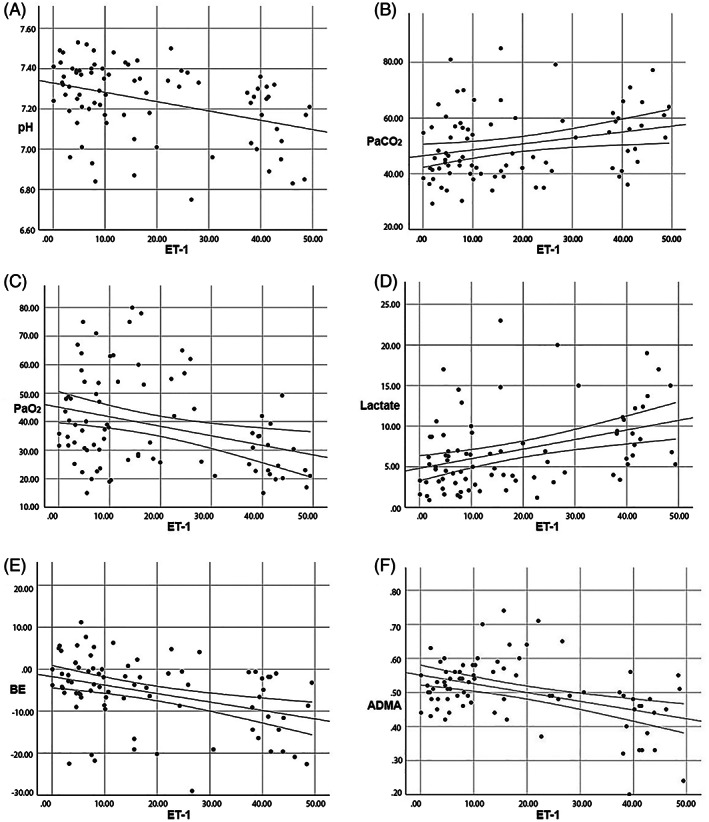
Linear regression analysis graphs between ET‐1 concentrations and pH (A), PaCO_2_ (B), PaO_2_ (C), BE (E), and ADMA (F) concentrations. ET‐1 concentrations were determined positive correlation PaCO_2_ (B) and lactate concentrations (D). ADMA, asymmetric dimethylarginine; BE, base deficit; ET‐1, endothelin‐1; Lac, lactate; PaCO_2_, arterial partial pressure of carbon dioxide; PaO_2_, arterial partial pressure of oxygen; SO_2_, oxygen saturation

**FIGURE 2:**
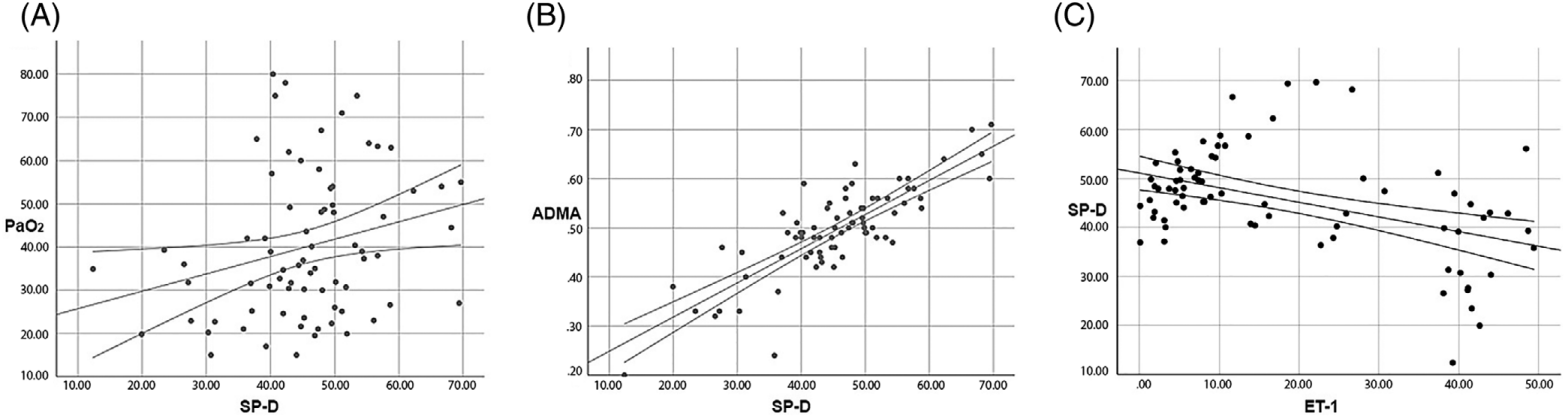
Linear regression analysis graph between SP‐D levels and PaO_2_, ADMA, and ET‐1 concentrations. A positive correlation between serum SP‐D concentrations and PaO_2_ (A) and ADMA (B) was determined whereas a negative correlation was found ET‐1 concentrations (C). ADMA, asymmetric dimethylarginine; ET‐1, endothelin‐1; PaO_2_, arterial partial pressure of oxygen; SP‐D, surfactant protein

Correlation results between some arterial blood gas parameters and ET‐1 concentrations in the premature and healthy calves are presented in Table 3 and Figure 1. A negative correlation between ADMA and ET‐1 concentrations (*P* < .01; Table 3; Figure 1) and a positive correlation with SP‐D concentrations (*P* < .01) were identified (Table 3; Figure 2). A negative correlation (*P* < .01) was found between ET‐1 and SP‐D concentrations (Figure 2).

**FIGURE 3:**
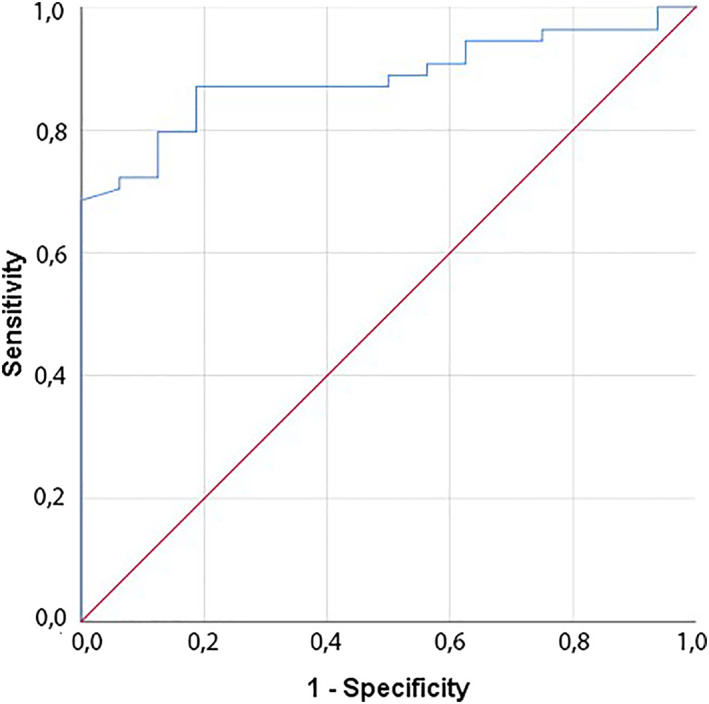
Receiver operator characteristic curve analysis graph based on serum ET‐1 concentration for surviving and dying premature calves. Performed ROC analysis to determine the relationship of ET‐1 concentration with mortality in premature calves determined that the ET‐1 concentration has prognostic importance to determining mortality. ET‐1, endothelin‐1; ROC, receiver operating characteristic curve

The results of the premature calves also showed that the concentrations of ET‐1 from the nonsurvivor calves were significantly higher than those of the survivor calves (*P* < .001; Table 4). Receiver operating characteristic curve (ROC) analysis for the utility of ET‐1 in differentiating between the survivor and nonsurvivor calves estimated an area under the curve (AUC) of 0.884 (*P* = .000, 95% confidence interval [CI], 0.806‐0.961; Table 5; Figure 3).

**TABLE 4:** Comparison of endothelin‐1 (ET‐1) concentrations in calves that died or survived (Median [min‐max])

Variable	Survivor (n: 54)	Nonsurvivor (n: 16)	*P* value
ET‐1(ng/L)	8.05 (0.08‐48.69)	39.58 (15.63‐49.40)	.000

**TABLE 5:** Importance of ET‐1 concentration in mortality prediction as a result of receiver operating characteristic curve (ROC) analysis

Variable	AUC	SE	*P* value	95% Cl		Cutoff value	Sensitivity (%)	Specificity (%)
				Lower limit	Upper limit			
ET‐1 (ng/L)	0.884	0.039	.000	0.806	0.961	34.04	87	82

Abbreviations: AUC, area under the curve, CI, confidence interval; ET‐1; endothelin‐1.

## 4 DISCUSSION

In our study, arterial blood pH, PaCO_2_, PaO_2_, SO_2_, BE, and lactate concentrations of calves were evaluated. The results indicate that pH, PaO_2_, SO_2_, and BE concentrations decreased and PaCO_2_ and lactate concentrations increased in premature calves with RDS compared to the control group whereas no differences were detected in non‐RDS premature calves. According to the results, arterial blood gas measurements were found to be reliable and accurate in determining whether RDS developed in premature calves. As many researchers have reported earlier,^7^, ^8^, ^27^, ^29^ our study also indicated that pH, PaCO_2_, PaO_2_, SO_2_, and lactate are the best blood gas parameters for the evaluation of lung function in premature calves with RDS.

In RDS caused by interstitial inflammation, excessive strain‐related PHT, interstitial edema, insufficient lung inflation and aeration, and development of hypoxia are the most important causes of mortality. These symptoms develop as a result of disrupted gas exchange with surfactant deficiency associated with pulmonary adaptation disorder caused by lack of lung development in premature calves.^7^, ^8^, ^27^ The causes of high mortality in premature calves with RDS have been reported to be severe hypercapnia and hypoxemia.^7^, ^8^, ^27^, ^30^, ^31^, ^32^ In our study, 15 (30%) of 50 premature calves with RDS died whereas only 1 calf (5%) of the 20 non‐RDS premature calves died, which suggests that RDS in the premature calves was the most important cause of mortality.

Earlier studies on premature calves with RDS have found variable severity of changes in blood gases and acid‐base balance.^7^, ^8^, ^31^ In addition to hypercapnia and hypoxia in premature calves with RDS, mixed acidosis (respiratory‐metabolic acidosis) is common.^3^, ^6^, ^8^ In our study, a positive correlation between pH and PaO_2_, SO_2_, and BE and a negative correlation between PaCO_2_ and lactate concentrations were observed.

Blood pH and BE are commonly accepted as valuable parameters for detecting mixed (respiratory‐metabolic acidosis) acidosis in calves after birth.^33^, ^34^ The decrease in blood pH occurs as a result of insufficient removal of CO_2_ from the lung and accumulation in the blood.^35^ In addition to PCO_2_, a high L‐lactate concentration also contributes to the development of acidosis.^7^, ^8^, ^36^ Lactate is produced under hypoxic conditions and poor tissue perfusion, and is used as an indirect marker of tissue hypoxia.^5^, ^7^, ^8^, ^37^, ^38^ A study in premature infants reported a strong correlation between lactate concentrations and BE and found that the effect of lactate on blood pH increased mortality rates.^39^ Base deficit often is used as an indirect indicator of lactic acidosis.^40^ Lactate plays an important role in the development of acidosis in newborns with asphyxia and is responsible for metabolic acidosis. It remains in the blood at high concentrations much longer than CO_2_.^7^, ^8^, ^36^ In our study, hypoxia, hypercapnia, a significant increase in lactate concentration, a decrease in BE, and acidosis were identified in premature calves with RDS, whereas the blood gas parameters of non‐RDS premature calves were within normal limits. Our study showed that significant changes occurred in blood gases and acid‐base balance in premature calves with RDS. Changes in blood gases such as an increase in blood PaCO_2_ and lactate concentrations and decreases in PaO_2_ and SO_2_ are the most important indicators of tissue hypoxia during the development of RDS.^4^, ^6^, ^8^, ^27^, ^31^, ^41^ Increased PaCO_2_ and lactate concentrations and decreased PaO_2_ and SO_2_ indicate widespread tissue hypoxia as a result of impaired lung function in premature calves with RDS and indicate severe respiratory acidosis in these cases.

In our study, GDF‐15, ADMA, ET‐1, VEGF, and SP‐D biomarkers were evaluated as indicators of lung endothelial and epithelial damage in the evaluation of PHT in premature calves with and without RDS and in comparison to normal healthy calves.

Endothelin‐1 is a peptide abundantly found in the human lung and has been reported to play an important role in the development of PHT because of the presence of endothelin receptors (ET‐A and ET‐B) on vascular smooth muscle cells.^13^ Endothelin‐1 concentration was found to increase significantly in cases of pulmonary hypertension.^15^, ^22^ A previous study reported higher concentrations of ET‐1 in cases of PHT compared to normal healthy individuals.^42^ On the other hand, it was determined that the mortality rate was higher in patients with PHT having high concentrations of ET‐1.^13^ In our study, serum ET‐1 concentrations of premature calves with RDS were significantly increased compared to those of the non‐RDS premature and control group calves. Although ET‐1 can be expressed in numerous tissues and organs, concentrations of ET‐1 mRNA were at least 5‐fold higher in lung than in any other organ.^43^ These findings suggest the development of PHT in premature calves with RDS as has been confirmed by previous studies in humans.^15^, ^22^, ^42^Also, serum ET‐1 concentrations of nonsurvivor premature calves were found to be higher than those of surviving premature calves (Table 4). Receiver operating characteristic curve analysis to determine the relationship of ET‐1 concentrations with mortality in premature calves determined that the ET‐1 had prognostic importance in determining mortality with a cutoff of 34 ng/L (Table 5). A previous study reported higher mortality rates in people with PHT with high concentrations of ET‐1,^12^ which is consistent with our hypothesis that RDS‐related PHT could develop in premature calves.

The correlation between blood gas parameters and ET‐1 also was evaluated. A negative correlation was found between ET‐1 concentrations and pH, PaO_2_, SO_2_, and BE, and a positive correlation between PaCO_2_ and lactate concentrations was observed. In conjunction with the development of PHT in lambs with experimentally induced RDS, plasma ET‐1 concentrations and PaCO_2_ increased and pH and PaO_2_ decreased in response to RDS.^8^ However, in infants with RDS, blood ET‐1 concentrations increased, the mechanism for which has not been fully elucidated. However, it was found in rats that increases in plasma and pulmonary ET‐1 concentrations are positively correlated with the severity of hypoxia and that ET‐1 plays a role in hypoxia‐related pulmonary arterial narrowing or PHT.^44^ In our study, correlations related to ET‐1 concentrations are consistent with those of earlier studies,^9^, ^44^ and we conclude that hypoxia and acidosis during RDS may cause ET‐1 synthesis and release into the bloodstream. The increase in serum ET‐1 concentration observed in premature calves with RDS may be related to the pulmonary vasoconstriction caused by hypoxia.

Nitric oxide (NO) is produced from L‐arginine by NO synthase and plays a central role in maintaining low pulmonary vascular resistance.^45^, ^46^, ^47^ Nitric oxide production is dependent on oxygen, and lack of NO synthesis under hypoxic conditions contributes to chronic hypoxic pulmonary vasoconstriction.^48^, ^49^ A significant increase in the concentration of ADMA occurred with endothelial damage.^17^ Increased ADMA concentrations cause a decrease in NO, which then increases vascular tone. Therefore, ADMA may be a useful biomarker in identifying PHT.^18^ A previous study determined that the secretion of ADMA decreased the activity of connexin 43, causing pulmonary endothelial dysfunction.^50^ An increase in the concentration of ADMA is considered to be an indicator of poor prognosis in patients with PHT, congestive heart failure, and portopulmonary hypertension and is negatively correlated with right atrial pressure and positively with venous oxygen saturation.^18^ Plasma ADMA concentrations were increased in infants with RDS.^51^ In a study conducted in premature infants with bronchopulmonary dysplasia, plasma ADMA concentrations were found to be higher in preterm infants with PAH than in infants without PAH.^14^ In PAH, the concentration of ET‐1, as a vasoconstrictor agent, increases and the concentration of NO, as a vasodilator agent, decreases. Endothelin‐1 concentrations in the circulation of PAH patients increase along with pulmonary vascular resistance.^43^ In our study, serum ADMA concentrations of the calves with RDS were decreased compared to those of the control group. Earlier studies^14^, ^51^ found a positive correlation between ADMA and ET‐1 concentrations, whereas we detected a negative correlation. Although our results contradict previous studies, given the central role of NO in maintaining low pulmonary vascular resistance, the low concentration of ADMA in premature calves with RDS was considered to be associated with stimulation of vasodilatation to decrease PAH or hypoxic pulmonary vasoconstriction by increasing NO production.

The main cause of RDS in prematurity is the lack of surfactant because of inadequate development of the lungs. Surfactant protein‐D is secreted by type II pneumocytes and plays an important role in maintaining the surface integrity of alveoli. Studies in infants with RDS determined that, in the first day of life, protein‐A and SP‐D concentrations were significantly lower than those of in healthy infants.^52^ Research examining genetic predisposition to RDS focused on genes for surfactant protein and found that the lack of SP‐D gene alleles in premature infants was associated with RDS development.^53^ In rabbits in which surfactant was experimentally removed from the lung, plasma and pulmonary ET‐1 concentrations were increased.^54^ Mice that lack SP‐D have increased NO production and inducible NO synthase (iNOS) expression in the bronchoalveolar (BAL) fluid.^55^ In patients with ARDS, BAL fluid SP‐D concentrations of nonsurvivors on day 1 were significantly lower than those of survivors and BAL SP‐D concentrations were associated with arterial‐to‐inspired oxygen (PaO_2_/FiO_2_) ratio on days 1 and 3 after the onset of ARDS. In other studies investigating ARDS, it was found that differentiation in SP‐D concentrations may provide important information about the prognosis of the disease.^16^, ^26^ It was found that SP‐D concentrations decreased after destruction of type II pneumocytes in the lungs and were closely related to the severity of damage to the lung.^25^

In our study, serum SP‐D concentrations were significantly lower in premature calves with RDS compared to the control group calves. A positive correlation between SP‐D concentrations and PaO_2_ and ADMA concentrations was determined whereas a negative correlation between SP‐D and ET‐1 concentrations was detected. The reason for the low concentration of SP‐D in the premature calves with RDS was thought to be related to inadequate development of the lungs and hypoxia‐induced destruction of type II pneumocytes. Previous results^25^ also support our hypothesis. Identification of a negative correlation between the biomarkers ET‐1 and SP‐D during lung damage confirms this conclusion.

Although our results were similar to those found in human medicine, some limitations still exist. The most important limiting factor in our study is that the presence PAH was not confirmed by invasive or noninvasive methods. Therefore, the biomarker findings of our study should be interpreted carefully until they are evaluated together with direct measurements of PAH.

## 5 CONCLUSION

Significant changes occurred in blood gases and acid‐base balance in premature calves with RDS. Serum ET‐1 concentrations were found to be high and ADMA and SP‐D concentrations were low in premature calves with RDS. Thus, serum ET‐1 may be useful in the diagnosis of PAH in premature calves with RDS, and a cutoff of 34 ng/mL corresponds to a sensitivity of 87% and a specificity of 82% for prediction of mortality.

## CONFLICT OF INTEREST DECLARATION

Authors declare no conflict of interest.

## OFF‐LABEL ANTIMICROBIAL DECLARATION

Authors declare no off‐label use of antimicrobials.

## INSTITUTIONAL ANIMAL CARE AND USE COMMITTEE (IACUC) OR OTHER APPROVAL DECLARATION

Approved by the Ethics Board of Selcuk University Veterinary Faculty Experimental Animals Production and Research Center (SÜVDAMEK) Ethics Board (approval number: 2019/79).

## HUMAN ETHICS APPROVAL DECLARATION

Authors declare human ethics approval was not needed for this study.
